# Frequency of *Chlamydia trachomatis* in Women with Cervicitis in Tehran, Iran

**DOI:** 10.1155/2007/67014

**Published:** 2007-02-05

**Authors:** Farhad B. Hashemi, Babak Pourakbari, Javad Zaeimi Yazdi

**Affiliations:** ^1^Department of Microbiology, School of Medicine, Tehran University of Medical Sciences, Tehran 14155, Iran; ^2^Infectious Disease Research Center, Tehran University of Medical Sciences, Tehran 14194, Iran; ^3^Department of Pathobiology, School of Medicine, Yazd University of Medical Sciences, Yazd 89195, Iran

## Abstract

*Chlamydia trachomatis* (CT) is the most common cause of bacterial sexually transmitted infection (STI) worldwide, but current data concerning the prevalence of CT among women in Iran is scarce. Data regarding the frequency of CT infection among Iranian women can help to justify the implementation of a national CT screening program that can reduce the high morbidity associated with sequelae of CT infections by treating infected women. Endocervical secretions from 123 married women (20–55 years) with cervicitis were tested by a PCR-EIA method using primers to amplify a CT-specific plasmid. The digoxigenin-labeled amplicon was measured by hybridization to a biotin-labeled probe and a strepavidin-coated plate, followed by an enzyme-linked colorimetric analysis. Overall frequency of CT infection among women was 17% (21/123). The range of CT frequency among various age groups was 12–25%. The 31–40-year-age group comprised the majority (49%) of CT positive samples, followed by 20–30 year group (33%). Although the 20-to-30-year-old women reported the highest frequency of STI history, they had the lowest relative frequency of CT infection (12%). There is a high frequency of CT infection among women with cervicitis in Tehran, Iran, thus indicating a necessity to implement a routine CT screening program in the major cities of Iran and possibly nationwide. Identification of CT-infected women may prevent its spread, and thereby reduce the high morbidity associated with CT infections among women in Iran.

## 1. INTRODUCTION

*Chlamydia trachomatis* (CT) is the most common cause of bacterial sexually transmitted infections (STI), with millions of cases reported annually throughout the world [1]. Most genital CT infections in women are asymptomatic; however, chlamydial infections can commonly lead to sequelae complications such as pelvic inflammatory disease (PID), an increased risk of ectopic pregnancy, and infertility [2, 3]. Early diagnosis is essential for the timely treatment of CT-infected women to prevent the development of sequelae and preventing the transmission of CT to susceptible individuals. National screening programs that identify and treat CT-infected women have been shown to reduce the rates of CT infections and the morbidity associated with CT infections [4, 5]. Regrettably, nationwide STI surveillance programs are absent in Iran, and a majority of women have inadequate access to reproductive health services or STI clinics. Consequently, information concerning the prevalence of CT among women in Iran is scarce. Current data regarding CT infection from a region is essential for controlling its spread and for helping assess the impact of CT on the reproductive health of women. In this small study, we report the incidence of CT infection among women with cervicitis in Tehran, Iran.

## 2. MATERIALS AND METHODS

### 2.1. Patients and specimens

A total of 123 married women (aged 20–55 years) with symptomatic cervicitis participated in this case series study. Participants visited the obstetrics and gynecology section of Mirza Kouchek Khan Hospital in Tehran, Iran, between December 2004 to June 2005, primarily complaining of pelvic pain and/or vaginal discharge. All women who received antibiotic treatment within three weeks prior to their visit were excluded from our study. Consenting participants completed a questionnaire before cervical examination in which they were asked for demographic data and any STI history (i.e., self-reported). Cervical examination included the evaluation for the presence of mucopurulent endocervical discharge, friability, and ectropin. After removing cervical mucus, samples of the endocervical canal secretions were collected on two cotton swabs, which were washed in 500 *μ*L of phosphate buffered saline and the fluid was stored at −20°C. DNA was extracted from 100 *μ*L of the endocervical sample fluid using the DIAtom Prep100 kit (IsoGene Inc., Moscow, Russia) and was stored at −20°C until used.

### 2.2. PCR amplification

This method has been described in detail elsewhere [6]. Briefly, a 377 bp fragment of a cryptic plasmid (specific to *C. trachomatis*) was amplified through 30 cycles (pre-denaturation step at 94°C 3.5 min, denaturation at 94°C 30 s, primer annealing at 52°C, and 30 s primer extension at 72°C 3 min) in 25 *μ*L PCR reaction buffer (consisting of 10 mmol/L TRIS, pH 8.3, 50 mmol/L KC1, 2.5 mmol/L MgCl_2_, and 0.01% gelatin); 0.2 mmol/L each of dATP, dGTP, dCTP, and dTTP; 2.5U *Taq* DNA polymerase; and 0.5 *μ*mol/L primers for conserved plasmid nucleotide sequences of *C. trachomatis* (Table 1).

### 2.3. PCR-EIA

The amplicon was measured by the detection of digoxigenin-labeled *C. trachomatis* PCR products in a microtiter-based enzyme immunoassay (PCR-EIA). Briefly, 10 *μ*L of amplicon was denatured with 20 *μ*L NaOH and incubated at room temperature (RT), and after 10 min, 220 *μ*L of hybridization buffer containing 10 pmol of the 5′ end biotin-labeled CT-specific internal oligonucleotide probe (complementary to the primers) was added. The amplicon was hybridized in solution for 30 min at 54°C, and the mixture was added to a strepavidin-coated 96-well microtiter plate. Using a hybridization-based EIA (Roche Diagnostics, Mannheim, Germany), 200 *μ*L of 1 : 100 diluted peroxidase-conjugated antidigoxigenin IgG was added to each well, incubated for 30 min at 37°C. After the wash (5X), 200 *μ*L of the ABTS substrate solution was added, and the plate was incubated for 30 min at 37°C. The optical density (OD) of the samples was measured by spectrophotometry at 405 nm absorbance (reference filter: 492 nm). The PCR-EIA run was considered valid if (−) control OD values were <0.1, and the PCR positive control value was >1.0. The background OD value was 0.055, calculated as the mean of all (−) control samples plus 3 standard deviations (SD). A sample was considered (+) if its OD value was greater than the background cutoff value by threefold (i.e., 0.165). The mean OD value of CT positive control samples was 1.81 ± 0.789.

## 3. RESULTS

Table 2 shows the rates of *C. trachomatis* infection among all four age groups of married women studied. Overall, 17% (21/123) of the specimens were positive for chlamydial DNA by the PCR-EIA assay. The CT infection frequency rate ranged from 12% to 25% among the patient age groups (Table 2). Although the 31–40-year group represented 35% of the samples, this age group comprised a marked 49% (10/21) of CT positive samples. Conversely, the 20–30-year group, which represented about half the patients investigated, comprised only 33% (7/21) of the *C. trachomatis* positive samples. Interestingly, when these two groups were combined, the 20–40-year group represented 81% of women in this study, and their samples comprised 81% of all CT positive specimens, as well.

On the other hand, as Table 2 demonstrates, when the CT positive samples were adjusted for the number of specimens within each age group of women, the highest rate of CT positive samples (25%) was from the >51-year-old women (1/4), however, the >51-year-age group represented <1% of the samples studied. The 31–40 age group showed the second highest rate of CT infection with 23.3% (10/43), followed by the 41–50-year-old women comprising 15.8% (3/19) of CT positive samples.

Overall, 29% (36/123) of patients reported to have STI history. Interestingly, even though the youngest group (20–30 years) in our study reported the highest adjusted rate of STI history 35% (20/57), these women showed the lowest (12.3%) adjusted frequency of CT infection with (Table 2). With the exception of the 31–40-year-age group, among all age groups investigated, the proportion of women who admitted to having a history of STI was higher than that of those with genital CT infection (Table 2).

## 4. DISCUSSION

The 17% frequency rate of CT genital infections among married women in Iran is in contrast with the 7% rate previously reported from Iran in the early 1980s [7]. Although the patients in neither study are representative of the general population, including asymptomatic women, the current CT infection rate is higher than the 4–11% prevalence rates reported from Slovenia, Holland, Colombia, Canada, and the United States [5, 8–11]. The difference in genital CT infection rate among women in this study as compared to recent reports from Tehran [6, 12] may relate to the improved sensitivity of the PCR-EIA employed, as well as the differences in the type of patients and specimens analyzed.

Our findings highlight the need for routine CT screening, particularly among Iranian women aged <40 years, for early detection of genital chlamydial infection to reduce the morbidity associated with sequelae. It has been shown that the level of chlamydial cervicitis decreases quickly in countries where routine screening for CT has become mandatory [4]. Early diagnosis and appropriate treatment of chlamydial cervicitis among the younger women will help to prevent transmission of *C. trachomatis* to susceptible individuals and avoid severe complications that eventually develop in older women. Initially, screening programs may focus on patients visiting the infertility or STI clinics in large cities of Iran and can later expand to cover the symptomatic as well as asymptomatic CT-infected women throughout the country.

We acknowledge that our results would have been enhanced by a larger sample size as well as additional demographic and behavioral data (i.e., socioeconomic status) which would have allowed for advanced data analysis. Overcoming obstacles, which hinder studies like ours, such as the social stigma associated with patients visiting the STD clinics plus increasing the number of women's health centers offering diagnostic services for management of CT cervicitis can make a significant impact on women's reproductive health by preventing the long-term sequelae associated with PID [3, 13]. Nevertheless, our data lends support to implementing a nationwide screening program to identify and treat Iranian women with CT infections using sensitive methods. Other countries have shown that such programs are cost-effective and can lead to reduced morbidity associated with chlamydial infections [14]. Since most women with genital chlamydial infection are asymptomatic, and our study did not include this group of women, the rate of CT infection might be higher than 17% among women in Tehran. Therefore, improving the status of women's reproductive health care services is well justified in the large cities of Iran.

To the best of our knowledge, this is the first report from Iran that provides current data regarding frequency of genital CT infection among married Iranian women with cervicitis, and may be particularly helpful to physicians treating such patients in Iran, where the quality of women's health care requires drastic improvement.

In conclusion, the high frequency of CT genital infections among married women in Iran warrants a comprehensive study to screen a large number of women suffering from chlamydial infection, thus allowing for better estimates of the real magnitude of the reservoir of asymptomatic CT infections among Iranian women. In light of studies that have shown chlamydial genital infections which can serve as biological cofactors for the transmission of the human immunodeficiency virus (HIV) [15–17], and a cofactor in human papillomavirus (HPV) infection [18], screening programs for genital chlamydial infections may reduce the morbidity associated with other sexual-transmitted pathogens in women and help decrease the cost of reproductive as well as general health care in developing countries, such as Iran.

## Figures and Tables

**Table 1:** The DNA sequence of PCR primers and the probe used for detection of *C. trachomatis* by PCR-EIA assay.

Primer/probe name	DNA sequence (5′ → 3′)

BP1 (sense)	AACCGTTTTTAATAGTGGCA
BP2 (antisense)	TTCTGGCCAAGAATTATCC
BP3 (probe)	AGCAGCTGCGAAAAAGAGAC

**Table 2:** Frequency of *C. trachomatis* infection among women with cervicitis according to their age groups. (CT = *C. trachomatis*; STI = sexually transmitted infection.)

Age group	Patient number	(+) STI history	(+) CT	% (+) CT	% (+) CT
(year)	(%)	number (%*)	number	(of total*)	(within each age group)

20–30	57 (47)	20 (16)	7	5.7%	12.3%
31–40	43 (35)	8 (7)	10	8.1%	23.3%
41–50	19 (15)	4 (3)	3	2.4%	15.8%
≥ 51	4 (3)	4 (3)	1	0.8%	25.0%

Total	123 (100)	36 (29)	21	17.0	—

77*Of total number of specimens studied (*N* = 123).

## References

[1] Gerbase AC, Rowley JT, Mertens TE (1998). Global epidemiology of sexually transmitted diseases. *The Lancet*.

[2] Paavonenl J, Eggert-Kruse W (1999). *Chlamydia trachomatis*: impact on human reproduction. *Human Reproduction Update*.

[3] Center for Disease Control Prevention (CDC) Sexually Transmitted Disease Surveillance Report.

[4] Miller WC, Ford CA, Morris M (2004). Prevalence of chlamydial and gonococcal infections among young adults in the United States. *Journal of the American Medical Association*.

[5] Kese D, Maticic M, Potocnik M (2005). *Chlamydia trachomatis* infections in heterosexuals attending sexually transmitted disease clinics in Slovenia. *Clinical Microbiology and Infection*.

[6] Fatollahzadeh B, Mirsalehian A, Kazemi B, Arshadi H, Pourakbari B (2004). Detection of *Chlamydia trachomatis* and *Neisseria gonorrhoeae* in first-void urine from patients with urethritis by multiplex PCR. *Journal School of Medicine*.

[7] Darougar S, Aramesh B, Gibson JA, Treharne JD, Jones BR (1983). Chlamydial genital infection in prostitutes in Iran. *British Journal of Venereal Diseases*.

[8] Munk C, Morris SA, Kjaer SK (1999). PCR-detected *chlamydia trachomatis* infections from the uterine cervix of young women from the general population: prevalence and risk determinants. *Sexually Transmitted Diseases*.

[9] Molano M, Weiderpass E, Posso H (2003). Prevalence and determinants of *Chlamydia trachomatis* infections in women from Bogota, Colombia. *Sexually Transmitted Infections*.

[10] Jolly AM, Moffatt MEK, Fast MV, Brunham RC (2005). Sexually transmitted disease thresholds in Manitoba, Canada. *Annals of Epidemiology*.

[11] Gershman KA, Barrow JC (1996). A tale of two sexually transmitted diseases: prevalences and predictors of chlamydia and gonorrhea in women attending Colorado family planning clinics. *Sexually Transmitted Diseases*.

[12] Fallah F, Kazemi B, Goudarzi H (2005). Detection of *Chlamydia trachomatis* from urine specimens by PCR in women with cervicitis. *Iranian Journal of Public Health*.

[13] Einwalter LA, Ritchie JM, Ault KA, Smith EM (2005). Gonorrhea and chlamydia infection among women visiting family planning clinics: racial variation in prevalence and predictors. *Perspectives on Sexual and Reproductive Health*.

[14] Marrazzo JM, Celum CL, Hillis SD, Fine D, Delisle S, Handsfield HH (1997). Performance and cost-effectiveness of selective screening criteria for *Chlamydia trachomatis* infection in women: implications for a national chlamydia control strategy. *Sexually Transmitted Diseases*.

[15] Laga M, Manoka A, Kivuvu M (1993). Non-ulcerative sexually transmitted diseases as risk factors for HIV-1 transmission in women: results from a cohort study. *AIDS*.

[16] Plummer FA, Simonsen JN, Cameron DW (1991). Cofactors in male-female sexual transmission of human immunodeficiency virus type 1. *Journal of Infectious Diseases*.

[17] Grosskurth H, Mosha F, Todd J (1995). Impact of improved treatment of sexually transmitted diseases on HIV infection in rural Tanzania: randomised controlled trial. *The Lancet*.

[18] Finan RR, Tamim H, Almawi WY (2002). Identification of *Chlamydia trachomatis* DNA in human papillomavirus (HPV) positive women with normal and abnormal cytology. *Archives of Gynecology and Obstetrics*.

